# Ivermectin and Malaria—Putting an Elderly Drug to a New Test

**DOI:** 10.4269/ajtmh.19-0889

**Published:** 2020-02-06

**Authors:** William C. Campbell

**Affiliations:** Drew University, Madison, New Jersey

At the end of the 19th century, the discoveries of Ronald Ross and Battista Grassi convinced the world that malaria is transmitted by mosquitoes. By 1917, Ross was writing in anger and in frustration (and in verse) because the news of vector transmission was not being quickly translated into malaria control—and, for once, Grassi might have been in some agreement with Ross. Clearly, the control of malaria was not going to be easy, and indeed, progress would continue to be slow, not just over a period of a couple of decades, but over the next century. Advances in biomedical technology were spectacular, research was brilliant and unremitting, vaccines were developed, new drugs were introduced, attempts were made to exploit epidemiological weaknesses, and the effectiveness of regional malaria control programs was clearly demonstrated. Yet the sheer magnitude and complexity of malaria as a global disease made sustained widespread control unattainable. In the following pages, however, science makes clear, through the Ivermectin Roadmap, that its practitioners do not give up.

Now, an old, or at least elderly, drug is being put to a new test. The deleterious effect of the ivermectin molecule on insects had been reported from the Merck Laboratories by 1980 and was widely confirmed by others. From experience in the use of ivermectin in human populations, we have evidence that ivermectin has a good effect on the health of worm-infected people who ingest it, but a bad effect on the health of mosquitoes that imbibe the blood of those people. The Ivermectin Roadmap describes a critically important assessment of whether such an observation can be translated into large-scale control of mosquito-borne disease.

We have long known that the difference between a medicine and a poison is dosage, and that dosage must express both amount and time. But a single dosage of a drug is here being asked to treat one kind of animal (mammal) while—at the same time, so to speak—treating a very different kind of animal (insect) to combat one kind of pathogen (helminth) by destroying it and to combat a very different kind of pathogen (protist) by blocking its transmission. That is breaking new ground indeed! Establishing the feasibility of the program will not just be a matter of pharmacodynamic efficacy, or just managerial efficiency. Safety is critical, and it will not just be a matter of human safety. It will be much grander than that; it will be a matter of ecological safety.

Those who will implement the Roadmap deserve the applause and good wishes of those of us who watch from the sidelines—not just because the enterprise is daring, but because it seeks to make feasible a program that could lift an ancient scourge and thereby do the world a world of good.

